# (4,4′-Dimethyl-2,2′-bipyridine-κ^2^ *N*,*N*′)(dimethyl sulfoxide-κ*O*)diiodidocadmium(II)

**DOI:** 10.1107/S1600536810012572

**Published:** 2010-04-10

**Authors:** Khadijeh Kalateh, Roya Ahmadi, Vahid Amani

**Affiliations:** a Islamic Azad University, Shahr-e-Rey Branch, Tehran, Iran

## Abstract

In the title compound, [CdI_2_(C_12_H_12_N_2_)(C_2_H_6_OS)], the Cd^II^ cation is coordinated by two N atoms from a dimethyl­bipyridine ligand, one O atom from a dimethyl sulfoxide mol­ecule and two I^−^ anions in a distorted trigonal–bipyramidal geometry. Intra­molecular C—H⋯O hydrogen bonding and inter­molecular π–π stacking between parallel pyridine rings [centroid–centroid distance = 3.658 (3) Å] are present in the crystal structure.

## Related literature

For metal complexes of 4,4′-dimethyl-2,2′-bipyridine, see: Ahmadi *et al.* (2008 ▶); Amani *et al.* (2009 ▶); Kalateh *et al.* (2008 ▶); Bellusci *et al.* (2008 ▶); Hojjat Kashani *et al.* (2008 ▶); Sakamoto *et al.* (2004 ▶); Sofetis *et al.* (2006 ▶); Willett *et al.* (2001 ▶); Yoshikawa *et al.* (2003 ▶); Yousefi *et al.* (2008 ▶).
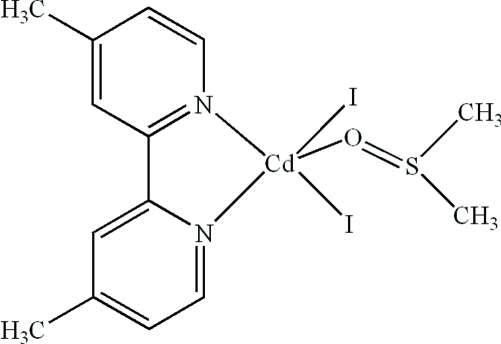


## Experimental

### 

#### Crystal data

[CdI_2_(C_12_H_12_N_2_)(C_2_H_6_OS)]
*M* *_r_* = 628.58
Monoclinic, 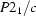

*a* = 8.729 (1) Å
*b* = 15.5247 (18) Å
*c* = 15.1354 (17) Å
β = 102.620 (9)°
*V* = 2001.5 (4) Å^3^
*Z* = 4
Mo *K*α radiation
μ = 4.28 mm^−1^
*T* = 298 K
0.49 × 0.30 × 0.28 mm

#### Data collection

Bruker SMART CCD diffractometer
Absorption correction: multi-scan (*SADABS*; Sheldrick, 1998 ▶) *T*_min_ = 0.002, *T*_max_ = 0.055
15568 measured reflections
5360 independent reflections
4625 reflections with *I* > 2σ(*I*)
*R*_int_ = 0.082

#### Refinement

*R*[*F* ^2^ > 2σ(*F* ^2^)] = 0.066
*wR*(*F* ^2^) = 0.172
*S* = 1.16
5360 reflections
195 parameters
H-atom parameters constrained
Δρ_max_ = 2.10 e Å^−3^
Δρ_min_ = −2.23 e Å^−3^

### 

Data collection: *SMART* (Bruker, 2007 ▶); cell refinement: *SAINT* (Bruker, 2007 ▶); data reduction: *SAINT*; program(s) used to solve structure: *SHELXS97* (Sheldrick, 2008 ▶); program(s) used to refine structure: *SHELXL97* (Sheldrick, 2008 ▶); molecular graphics: *ORTEP-3 for Windows* (Farrugia, 1997 ▶); software used to prepare material for publication: *WinGX* (Farrugia, 1999 ▶).

## Supplementary Material

Crystal structure: contains datablocks I, global. DOI: 10.1107/S1600536810012572/xu2734sup1.cif

Structure factors: contains datablocks I. DOI: 10.1107/S1600536810012572/xu2734Isup2.hkl

Additional supplementary materials:  crystallographic information; 3D view; checkCIF report

## Figures and Tables

**Table 1:** Selected bond lengths (Å)

Cd1—N1	2.366 (5)
Cd1—N2	2.326 (4)
Cd1—O1	2.313 (5)
Cd1—I1	2.7535 (6)
Cd1—I2	2.7674 (6)

**Table 2:** Hydrogen-bond geometry (Å, °)

*D*—H⋯*A*	*D*—H	H⋯*A*	*D*⋯*A*	*D*—H⋯*A*
C12—H12⋯O1	0.93	2.47	3.063 (8)	122

**Fig. 1.**
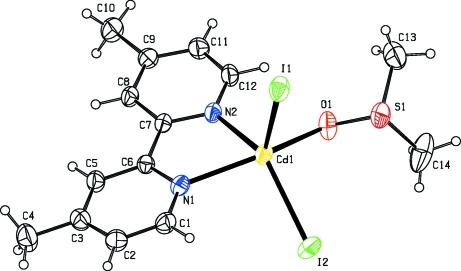
The molecular structure of the title molecule with the atom-numbering scheme. Displacement ellipsoids are drawn at the 30% probability level.

**Fig. 2.**
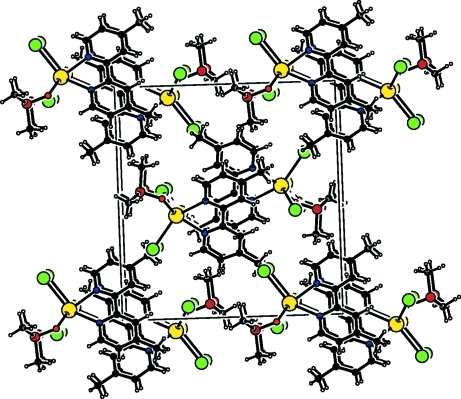
Unit-cell packing diagram for (I).

## supplementary crystallographic information

### Comment

4,4'-Dimethyl-2,2'-bipyridine (4,4'-dmbipy), is a good bidentate ligand, and numerous complexes with 4,4'-dmbipy have been prepared, such as that of mercury (Kalateh *et al.*, 2008; Yousefi *et al.*, 2008), indium (Ahmadi *et al.*, 2008), iron (Amani *et al.*, 2009), platin (Hojjat Kashani *et al.*, 2008), manganese (Sakamoto *et al.*, 2004), silver (Bellusci *et al.*, 2008), gallium (Sofetis *et al.*, 2006), copper (Willett *et al.*, 2001) and iridium (Yoshikawa *et al.*, 2003). Here, we report the synthesis and structure of the title compound.

In the title compound (Fig. 1), the Cd^II^ atom is five-coordinated in a distorted square-pyramidal configuration by two N atoms from one 4,4'-dimethyl-2,2'-bipyridine, one O atom from one dimethyl sulfoxide and two I atoms. The Cd—I and Cd—N bond lengths and angles are collected in Table 1.

In the crystal structure, intermolecular C—H···O hydrogen bonds (Table 2) and π-π contacts (Fig. 2) between the pyridine rings, Cg3—Cg2^i^ and Cg3—Cg3^ii^ [symmetry cods: (i) 2-X,2-Y,2-Z and (ii) 1-X,2-Y,2-Z , where Cg2 and Cg3 are centroids of the rings (N1/C1—C3/C5—C6) and (N2/C7—C9/C11—C12), respectively] may stabilize the structure, with centroid-centroid distance of 3.657 (3) and 3.775 (3) Å.

### Experimental

For the preparation of the title compound a solution of 4,4'-dimethyl-2,2'-bipyridine (0.15 g, 0.80 mmol) in methanol (10 ml) was added to a solution of CdI_2_ (0.29 g, 0.80 mmol) in methanol (5 ml) at room temperature. The suitable crystals for X-ray diffraction experiment were obtained by methanol diffusion to a colorless solution in DMSO. Suitable crystals were isolated after one week (yield; 0.36 g, 71.6%).

### Refinement

All H atoms were positioned geometrically with C—H = 0.93 (aromatic) and 0.96 Å (methyl) and constrained to ride on their parent atoms, with U_iso_(H)=1.2U_eq_(c).

### Crystal data

**Table:**

[CdI_2_(C_12_H_12_N_2_)(C_2_H_6_OS)]	*F*(000) = 1176
*M**_r_* = 628.58	*D*_x_ = 2.086 Mg m^−^^3^
Monoclinic, *P*2_1_/*c*	Mo *K*α radiation, λ = 0.71073 Å
Hall symbol: -P 2ybc	Cell parameters from 887 reflections
*a* = 8.729 (1) Å	θ = 1.9–29.3°
*b* = 15.5247 (18) Å	µ = 4.28 mm^−^^1^
*c* = 15.1354 (17) Å	*T* = 298 K
β = 102.620 (9)°	Block, colorless
*V* = 2001.5 (4) Å^3^	0.49 × 0.30 × 0.28 mm
*Z* = 4	

### Data collection

**Table:**

Bruker SMART CCD diffractometer	5360 independent reflections
Radiation source: fine-focus sealed tube	4625 reflections with *I* > 2σ(*I*)
graphite	*R*_int_ = 0.082
φ and ω scans	θ_max_ = 29.2°, θ_min_ = 1.9°
Absorption correction: multi-scan (*SADABS*; Sheldrick, 1998)	*h* = −11→11
*T*_min_ = 0.002, *T*_max_ = 0.055	*k* = −21→20
15568 measured reflections	*l* = −20→19

### Refinement

**Table:**

Refinement on *F*^2^	Secondary atom site location: difference Fourier map
Least-squares matrix: full	Hydrogen site location: inferred from neighbouring sites
*R*[*F*^2^ > 2σ(*F*^2^)] = 0.066	H-atom parameters constrained
*wR*(*F*^2^) = 0.172	*w* = 1/[σ^2^(*F*_o_^2^) + (0.0903*P*)^2^ + 1.8883*P*] where *P* = (*F*_o_^2^ + 2*F*_c_^2^)/3
*S* = 1.16	(Δ/σ)_max_ = 0.015
5360 reflections	Δρ_max_ = 2.10 e Å^−^^3^
195 parameters	Δρ_min_ = −2.23 e Å^−^^3^
0 restraints	Extinction correction: *SHELXL97* (Sheldrick, 2008), Fc^*^=kFc[1+0.001xFc^2^λ^3^/sin(2θ)]^-1/4^
Primary atom site location: structure-invariant direct methods	Extinction coefficient: 0.0171 (10)

### Special details

**Table:**

Geometry. All esds (except the esd in the dihedral angle between two l.s. planes) are estimated using the full covariance matrix. The cell esds are taken into account individually in the estimation of esds in distances, angles and torsion angles; correlations between esds in cell parameters are only used when they are defined by crystal symmetry. An approximate (isotropic) treatment of cell esds is used for estimating esds involving l.s. planes.
Refinement. Refinement of *F*^2^ against ALL reflections. The weighted *R*-factor wR and goodness of fit *S* are based on *F*^2^, conventional *R*-factors *R* are based on *F*, with *F* set to zero for negative *F*^2^. The threshold expression of *F*^2^ > σ(*F*^2^) is used only for calculating *R*-factors(gt) etc. and is not relevant to the choice of reflections for refinement. *R*-factors based on *F*^2^ are statistically about twice as large as those based on *F*, and *R*- factors based on ALL data will be even larger.

### Fractional atomic coordinates and isotropic or equivalent isotropic displacement parameters (Å^2^)

**Table:**

	*x*	*y*	*z*	*U*_iso_*/*U*_eq_	
C1	0.9509 (8)	0.7934 (4)	0.8795 (4)	0.0575 (14)	
H1	0.9529	0.7743	0.8216	0.069*	
C2	1.0322 (8)	0.7476 (4)	0.9527 (5)	0.0596 (14)	
H2	1.0897	0.6991	0.9439	0.071*	
C3	1.0280 (8)	0.7741 (4)	1.0397 (4)	0.0584 (14)	
C4	1.1139 (11)	0.7258 (6)	1.1206 (6)	0.082 (2)	
H4C	1.1322	0.7630	1.1725	0.098*	
H4B	1.2126	0.7060	1.1101	0.098*	
H4A	1.0522	0.6773	1.1313	0.098*	
C5	0.9435 (7)	0.8469 (4)	1.0476 (4)	0.0499 (12)	
H5	0.9383	0.8666	1.1049	0.060*	
C6	0.8651 (5)	0.8918 (3)	0.9714 (3)	0.0385 (9)	
C7	0.7726 (5)	0.9709 (3)	0.9790 (3)	0.0385 (9)	
C8	0.7692 (6)	1.0072 (4)	1.0620 (3)	0.0453 (10)	
H8	0.8262	0.9825	1.1151	0.054*	
C9	0.6794 (7)	1.0810 (4)	1.0656 (4)	0.0488 (11)	
C10	0.6735 (10)	1.1201 (5)	1.1561 (4)	0.0679 (17)	
H10A	0.7188	1.1767	1.1603	0.081*	
H10B	0.7315	1.0846	1.2037	0.081*	
H10C	0.5663	1.1239	1.1617	0.081*	
C11	0.5989 (8)	1.1146 (4)	0.9857 (4)	0.0568 (13)	
H11	0.5372	1.1635	0.9857	0.068*	
C12	0.6086 (8)	1.0764 (4)	0.9054 (4)	0.0545 (13)	
H12	0.5546	1.1013	0.8517	0.065*	
C13	0.411 (3)	1.1614 (9)	0.6212 (9)	0.189 (11)	
H13A	0.3652	1.1675	0.6730	0.227*	
H13B	0.3365	1.1789	0.5677	0.227*	
H13C	0.5029	1.1971	0.6287	0.227*	
C14	0.2811 (10)	1.0103 (11)	0.5584 (6)	0.110 (4)	
H14C	0.2942	0.9510	0.5437	0.132*	
H14B	0.2383	1.0418	0.5040	0.132*	
H14A	0.2108	1.0141	0.5989	0.132*	
N1	0.8687 (5)	0.8647 (3)	0.8888 (3)	0.0471 (9)	
N2	0.6925 (5)	1.0048 (3)	0.9006 (3)	0.0439 (9)	
O1	0.4994 (6)	1.0216 (4)	0.7072 (3)	0.0664 (12)	
Cd1	0.72703 (5)	0.94561 (3)	0.76515 (2)	0.04529 (16)	
I1	0.93442 (5)	1.04931 (3)	0.70096 (3)	0.06676 (19)	
I2	0.63195 (7)	0.80423 (3)	0.65550 (3)	0.0758 (2)	
S1	0.46338 (19)	1.05399 (12)	0.61041 (10)	0.0574 (4)	

### Atomic displacement parameters (Å^2^)

**Table:**

	*U*^11^	*U*^22^	*U*^33^	*U*^12^	*U*^13^	*U*^23^
C1	0.068 (3)	0.057 (3)	0.046 (3)	0.009 (3)	0.008 (2)	−0.013 (2)
C2	0.066 (3)	0.056 (3)	0.055 (3)	0.014 (3)	0.010 (3)	−0.008 (3)
C3	0.062 (3)	0.055 (3)	0.056 (3)	0.010 (3)	0.007 (3)	0.003 (3)
C4	0.094 (5)	0.082 (5)	0.063 (4)	0.032 (4)	0.006 (4)	0.008 (4)
C5	0.055 (3)	0.054 (3)	0.038 (2)	0.008 (2)	0.005 (2)	0.000 (2)
C6	0.038 (2)	0.042 (2)	0.036 (2)	−0.0003 (16)	0.0086 (16)	0.0007 (18)
C7	0.037 (2)	0.045 (2)	0.033 (2)	−0.0025 (17)	0.0059 (16)	−0.0002 (18)
C8	0.054 (3)	0.050 (3)	0.031 (2)	0.003 (2)	0.0069 (18)	−0.0019 (19)
C9	0.055 (3)	0.048 (3)	0.045 (3)	0.002 (2)	0.013 (2)	−0.003 (2)
C10	0.090 (5)	0.070 (4)	0.046 (3)	0.011 (4)	0.019 (3)	−0.009 (3)
C11	0.068 (3)	0.051 (3)	0.050 (3)	0.013 (3)	0.010 (3)	−0.004 (2)
C12	0.069 (3)	0.055 (3)	0.036 (2)	0.014 (3)	0.002 (2)	0.002 (2)
C13	0.38 (3)	0.084 (8)	0.079 (7)	0.051 (13)	−0.012 (12)	0.009 (6)
C14	0.061 (4)	0.200 (13)	0.063 (5)	−0.027 (6)	0.001 (4)	−0.007 (7)
N1	0.050 (2)	0.053 (2)	0.038 (2)	−0.0004 (18)	0.0086 (17)	−0.0057 (18)
N2	0.048 (2)	0.048 (2)	0.0333 (18)	0.0019 (17)	0.0039 (16)	−0.0008 (17)
O1	0.059 (2)	0.095 (4)	0.042 (2)	0.014 (2)	0.0060 (18)	0.010 (2)
Cd1	0.0492 (2)	0.0532 (3)	0.0323 (2)	−0.00441 (14)	0.00650 (14)	−0.00210 (14)
I1	0.0659 (3)	0.0866 (4)	0.0452 (2)	−0.0248 (2)	0.00653 (18)	0.01257 (19)
I2	0.0940 (4)	0.0675 (3)	0.0581 (3)	−0.0181 (2)	−0.0001 (2)	−0.0193 (2)
S1	0.0532 (7)	0.0810 (11)	0.0364 (6)	0.0013 (6)	0.0061 (5)	−0.0011 (6)

### Geometric parameters (Å, °)

**Table:**

C1—N1	1.342 (8)	C10—H10B	0.9600
C1—C2	1.376 (9)	C10—H10C	0.9600
C1—H1	0.9300	C11—C12	1.373 (8)
C2—C3	1.387 (9)	C11—H11	0.9300
C2—H2	0.9300	C12—N2	1.342 (8)
C3—C5	1.368 (8)	C12—H12	0.9300
C3—C4	1.491 (9)	C13—S1	1.746 (13)
C4—H4C	0.9600	C13—H13A	0.9600
C4—H4B	0.9600	C13—H13B	0.9600
C4—H4A	0.9600	C13—H13C	0.9600
C5—C6	1.393 (7)	C14—S1	1.751 (9)
C5—H5	0.9300	C14—H14C	0.9600
C6—N1	1.327 (6)	C14—H14B	0.9600
C6—C7	1.487 (7)	C14—H14A	0.9600
C7—N2	1.346 (6)	Cd1—N1	2.366 (5)
C7—C8	1.384 (7)	Cd1—N2	2.326 (4)
C8—C9	1.396 (8)	O1—S1	1.515 (5)
C8—H8	0.9300	Cd1—O1	2.313 (5)
C9—C11	1.363 (9)	Cd1—I1	2.7535 (6)
C9—C10	1.508 (8)	Cd1—I2	2.7674 (6)
C10—H10A	0.9600		
			
N1—C1—C2	122.4 (6)	C9—C11—H11	120.0
N1—C1—H1	118.8	C12—C11—H11	120.0
C2—C1—H1	118.8	N2—C12—C11	123.1 (5)
C1—C2—C3	119.6 (6)	N2—C12—H12	118.5
C1—C2—H2	120.2	C11—C12—H12	118.5
C3—C2—H2	120.2	S1—C13—H13A	109.5
C5—C3—C2	117.0 (6)	S1—C13—H13B	109.5
C5—C3—C4	121.8 (6)	H13A—C13—H13B	109.5
C2—C3—C4	121.1 (6)	S1—C13—H13C	109.5
C3—C4—H4C	109.5	H13A—C13—H13C	109.5
C3—C4—H4B	109.5	H13B—C13—H13C	109.5
H4C—C4—H4B	109.5	S1—C14—H14C	109.5
C3—C4—H4A	109.5	S1—C14—H14B	109.5
H4C—C4—H4A	109.5	H14C—C14—H14B	109.5
H4B—C4—H4A	109.5	S1—C14—H14A	109.5
C3—C5—C6	121.2 (5)	H14C—C14—H14A	109.5
C3—C5—H5	119.4	H14B—C14—H14A	109.5
C6—C5—H5	119.4	C6—N1—C1	118.9 (5)
N1—C6—C5	120.9 (5)	C6—N1—Cd1	117.5 (4)
N1—C6—C7	117.4 (4)	C1—N1—Cd1	123.6 (4)
C5—C6—C7	121.8 (4)	C12—N2—C7	117.5 (5)
N2—C7—C8	122.1 (5)	C12—N2—Cd1	123.5 (3)
N2—C7—C6	116.2 (4)	C7—N2—Cd1	118.7 (3)
C8—C7—C6	121.7 (4)	S1—O1—Cd1	121.0 (3)
C7—C8—C9	119.6 (5)	O1—Cd1—N2	82.36 (16)
C7—C8—H8	120.2	O1—Cd1—N1	145.92 (16)
C9—C8—H8	120.2	N2—Cd1—N1	70.02 (16)
C11—C9—C8	117.7 (5)	O1—Cd1—I1	98.31 (14)
C11—C9—C10	122.5 (6)	N2—Cd1—I1	107.57 (12)
C8—C9—C10	119.8 (5)	N1—Cd1—I1	108.61 (12)
C9—C10—H10A	109.5	O1—Cd1—I2	93.25 (14)
C9—C10—H10B	109.5	N2—Cd1—I2	139.68 (12)
H10A—C10—H10B	109.5	N1—Cd1—I2	95.15 (12)
C9—C10—H10C	109.5	I1—Cd1—I2	112.72 (2)
H10A—C10—H10C	109.5	O1—S1—C13	103.3 (5)
H10B—C10—H10C	109.5	O1—S1—C14	106.4 (5)
C9—C11—C12	120.0 (6)	C13—S1—C14	100.5 (9)
			
N1—C1—C2—C3	1.3 (11)	C8—C7—N2—C12	0.5 (8)
C1—C2—C3—C5	−1.2 (11)	C6—C7—N2—C12	−179.7 (5)
C1—C2—C3—C4	179.6 (8)	C8—C7—N2—Cd1	174.2 (4)
C2—C3—C5—C6	0.2 (10)	C6—C7—N2—Cd1	−6.0 (6)
C4—C3—C5—C6	179.4 (7)	S1—O1—Cd1—N2	152.1 (4)
C3—C5—C6—N1	0.8 (9)	S1—O1—Cd1—N1	−172.4 (3)
C3—C5—C6—C7	179.9 (6)	S1—O1—Cd1—I1	45.3 (4)
N1—C6—C7—N2	4.0 (7)	S1—O1—Cd1—I2	−68.2 (4)
C5—C6—C7—N2	−175.1 (5)	C12—N2—Cd1—O1	−22.7 (5)
N1—C6—C7—C8	−176.1 (5)	C7—N2—Cd1—O1	164.0 (4)
C5—C6—C7—C8	4.8 (8)	C12—N2—Cd1—N1	177.6 (5)
N2—C7—C8—C9	0.4 (8)	C7—N2—Cd1—N1	4.3 (4)
C6—C7—C8—C9	−179.4 (5)	C12—N2—Cd1—I1	73.7 (5)
C7—C8—C9—C11	−0.4 (9)	C7—N2—Cd1—I1	−99.6 (4)
C7—C8—C9—C10	179.2 (6)	C12—N2—Cd1—I2	−108.7 (5)
C8—C9—C11—C12	−0.5 (10)	C7—N2—Cd1—I2	78.0 (4)
C10—C9—C11—C12	180.0 (7)	C6—N1—Cd1—O1	−39.9 (6)
C9—C11—C12—N2	1.5 (11)	C1—N1—Cd1—O1	139.8 (5)
C5—C6—N1—C1	−0.8 (8)	C6—N1—Cd1—N2	−2.0 (4)
C7—C6—N1—C1	−179.9 (5)	C1—N1—Cd1—N2	177.7 (5)
C5—C6—N1—Cd1	179.0 (4)	C6—N1—Cd1—I1	100.5 (4)
C7—C6—N1—Cd1	−0.2 (6)	C1—N1—Cd1—I1	−79.8 (5)
C2—C1—N1—C6	−0.3 (10)	C6—N1—Cd1—I2	−143.5 (4)
C2—C1—N1—Cd1	−180.0 (5)	C1—N1—Cd1—I2	36.2 (5)
C11—C12—N2—C7	−1.4 (10)	Cd1—O1—S1—C13	−131.6 (9)
C11—C12—N2—Cd1	−174.8 (5)	Cd1—O1—S1—C14	123.1 (6)

### Hydrogen-bond geometry (Å, °)

**Table:**

*D*—H···*A*	*D*—H	H···*A*	*D*···*A*	*D*—H···*A*
C12—H12···O1	0.93	2.47	3.063 (8)	122
